# Candy Wrapper for the Earth's Inner Core

**DOI:** 10.1038/srep02096

**Published:** 2013-06-28

**Authors:** M. Mattesini, A. B. Belonoshko, H. Tkalčić, E. Buforn, A. Udías, R. Ahuja

**Affiliations:** 1 Departamento de Física de la Tierra, Astronomía y Astrofísica I, Universidad Complutense de Madrid, E-28040 Madrid, Spain; 2 Instituto de Geociencias (UCM-CSIC), Facultad de Ciencias Físicas, Plaza de Ciencias 1, 28040-Madrid, Spain; 3 Condensed Matter Theory, Department of Theoretical Physics, AlbaNova University Center, KTH Royal Institute of Technology, SE-10691 Stockholm, Sweden; 4 Research School of Earth Sciences, The Australian National University, Canberra, 0200 ACT, Australia; 5 Condensed Matter Theory Group, Department of Physics and Astronomy, Uppsala University, Box 516, 75120 Uppsala, Sweden; 6 Applied Materials Physics, Department of Materials Science and Engineering, Royal Institute of Technology, SE-10044 Stockholm, Sweden

## Abstract

Recent global expansion of seismic data motivated a number of seismological studies of the Earth's inner core that proposed the existence of increasingly complex structure and anisotropy. In the meantime, new hypotheses of dynamic mechanisms have been put forward to interpret seismological results. Here, the nature of hemispherical dichotomy and anisotropy is re-investigated by bridging the observations of PKP(bc-df) differential travel-times with the iron *bcc*/*hcp* elastic properties computed from *first-principles* methods.The *Candy Wrapper* velocity model introduced here accounts for a dynamic picture of the inner core (i.e., the eastward drift of material), where different iron crystal shapes can be stabilized at the two hemispheres. We show that seismological data are best explained by a rather complicated, mosaic-like, structure of the inner core, where well-separated patches of different iron crystals compose the anisotropic western hemispherical region, and a conglomerate of almost indistinguishable iron phases builds-up the weakly anisotropic eastern side.

The centre of the Earth is one of the most inaccessible and enigmatic parts of our planet. The full knowledge of its chemical composition, dynamics, anisotropic properties and hemispherical asymmetry in absolute velocity and attenuation is in the centre of long-standing and ongoing controversy^1^,^2^,^3^. This almost spherical solid body, with a radius of 1220 km, has been developing since more than one billion years ago, involving the process of crystallization of iron from the liquid outer-core. The present-day growing rate has been computed to be around 0.5 mm/year^4^. The underlying mineral physics and geophysical views about the solid inner core can thus be thought of as a narrow-sighted snapshot over the core's entire lifetime period. Solidification of the Earth's inner core (EIC) at the liquid-solid interface, also called the inner-core boundary (ICB), occurs under shear compression, a physicochemical process that indeed favors the development of axially oriented iron crystals. Although a number of mechanisms have been introduced to interpret the inner-core anisotropy^5^, most of them tightly relied on the lattice preferred orientation (LPO) of both hexagonal-close-packed (*hcp*)^6^,^7^ and the body-centered-cubic (*bcc*) iron crystals^8^,^9^,^10^. Cylindrical anisotropy of the order of 3% with the fast axis parallel to the rotation axis and the slow axis in the equatorial plane was the preferred explanation of early seismic observations^11^,^12^. However, with the expansion of seismological stations around the globe and the inclusion of new ray-paths^13^ traversing the EIC, interpretations about its anisotropic structure have become more complex. For example, a uniform inner core cylindrical anisotropy does not predict well the scattered PKPbc-PKPdf (Fig. 1) travel-time residuals associated with different polar channels (ξ< 35°)^14^. Apart from focusing on anisotropy, over the last 15 years, numerous seismological studies of EIC have revealed an east-west dichotomy in compressional velocity^15^,^16^,^17^, and seismic attenuation^18^,^19^ that has recently been interpreted by a lopsided growth^20^,^21^. According to this model, the western hemisphere is crystallizing and the eastern hemisphere is melting. This process includes an eastward translation of the inner core material that induces a translational thermal convection. Crystal size in the quasi-eastern hemisphere might be larger than typical wavelengths of body waves sensitive to EIC structure and this can explain observed lack of strong back-scattering^22^,^23^, while the melting component can explain higher attenuation.

Despite recent efforts to reconcile dynamical models with seismological observations, a bridge between these two disciplines and mineral physics has not yet been established. Hence there is now an increasing need for a more quantitative link between dynamical models and seismological observations, which requires understanding of the stable mineralogical iron phase at inner core conditions and its elastic properties. Here we show that a mosaic-type distribution of various iron phases, each of them including a different degree and type of lattice preferred orientation, can explain the complex elastic behavior of the EIC observed in seismological data. We obtain an EIC distribution map of the various iron phases, thus uniquely addressing from a mineral physics point of view the seismologically observed hemispherical dichotomy. While we show that this distribution agrees with the recently proposed convective translational model of the EIC, we acknowledge that it does not discard alternative models with asymmetric or more complex structure in the inner core.

## Results

### Projecting the complex seismic scenario into a mineral physics context

**Figure 2:**
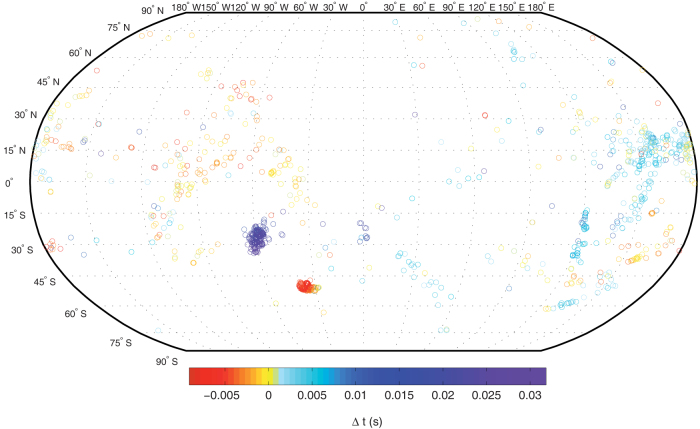
“Fast” and “slow” hemispheres of the Earth's inner core. Latitudes and longitudes of the symbols refer to piercing points of the compressional PKP waves at the Earth's inner core boundary. The seismologically inferred hemispherical dichotomy is revealed through negative Δt values (i.e, V_p _slower than the reference) in the quasi-western, and positive Δt values (i.e, V_p_ faster than the reference) in the quasi-eastern hemisphere.

We use 1117 high-quality PKP(bc-df) differential travel-times containing polar paths from both Antarctic and Alaskan stations^24^,^13^. The employed seismic spatial EIC coverage is shown in Fig. 2. All residuals are calculated with respect to the spherical Earth model *ak135*^25^ and corrected for ellipticity. The EIC is generally well sampled along equatorial paths by PKP waves due to the large number of favorable source-receiver configurations. Contrary to that, the spatial coverage along polar paths is relatively sparse due to the lack of large earthquakes and seismic stations located at extreme geographic latitudes. Such an inadequate sampling of polar paths has seriously hampered further progress on understanding EIC anisotropy. Apart from being exiguous, the available polar paths are also rather controversial^26^. For instance, the South Sandwich Islands (SSI) to Alaska sampling direction gives the most anomalous travel-time residuals and forms the basis for models alternative to inner core anisotropy models^2^. If we neglect the SSI data and consider only the differential travel-times residuals from the northern hemisphere to Antarctica stations, the strength of hypothetical uniform cylindrical anisotropy would be reduced to only 0.7%, however such a model is not uniquely required by the data^13^.

By inspecting seismological data, it has become rather clear that a simple anisotropic model cannot explain the complex behavior of travel-times^14^. Seismologically, there have also been observations of sharp velocity gradients, such as those under Central America^27^ and under the Indian Ocean^28^, and transitions between the two zones with different anisotropic properties. This tangled behavior is further sustained by the very similar theoretical melting temperatures computed for various Fe-model phases^29^. Therefore, when trying to translate the seismic scenario into a mineral physics context, one might assume that the structure of the EIC is made, to a first approximation, of patches of different iron crystals. Specifically, we consider the elastic behavior of the two most likely iron shapes (i.e. Fe-*hcp*^30^ and Fe-*bcc*^31^) to exist in the EIC, and explore their different axial orientations with respect to the Earth's rotational axis. The selected searching procedure is based on finding the Fe crystal orientation that best accounts for the available seismological travel-time data. That is, in each model, crystals of iron were spatially oriented in such a way that the resulting theoretical velocity curve comes to the best agreement with the observed differential travel-time residuals. The Earth's Cartesian coordinate system (*x*, *y* and *z*) and the crystallographic axes (*a*, *b* and *c*) are used throughout this paper to define the relative spatial orientation of the iron crystals with respect to the EIC.

### Selecting the polycrystalline iron crystal models and their spatial orientations

**Figure 3:**
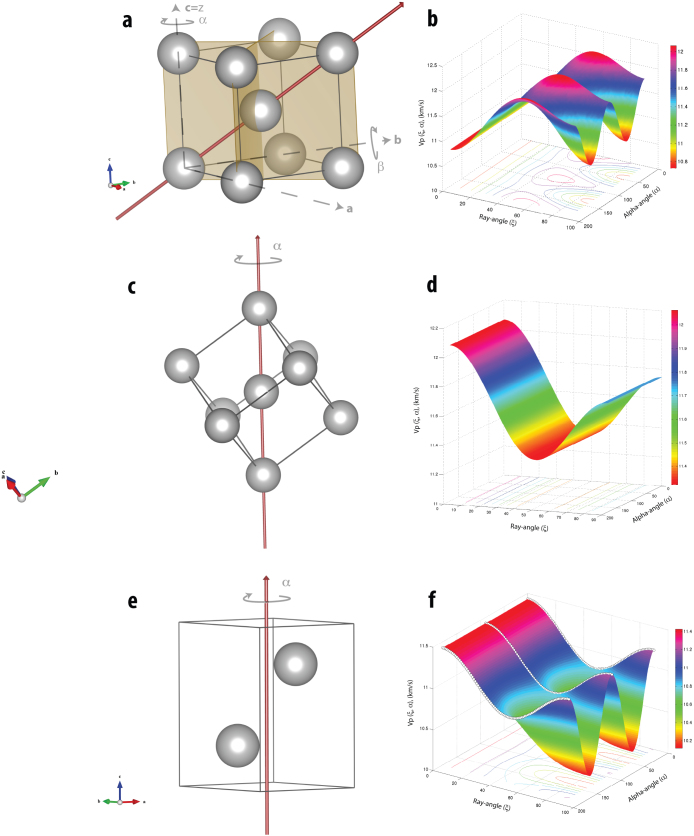
Three different kinds of velocity models. (a) Fe-*bcc* unit cell with one of the main diagonal directions [1 1 1] (red vector) representing the fast velocity axis. The two fast velocity planes [1 0 -1] and [1 1 0] at 90.0° with respect to each other are also shown in brown color. The α and β angles are clockwise rotations about the *c*- and *b*-axis, respectively. (b) Fe-*bcc* model, having its fast velocity axis at 54.74° from the main *z*-axis (α = β = 0°). This figure shows the periodicity of V_p_ produced by a clockwise rotation about the Earth's spin axis (i.e. α = 0°…180°). (c) 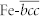 unit cell along the vertical crystallographic *c*-axis, aligned parallelly to the Earth's rotational z-axis. (d) 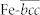 model, having its fast velocity axis (i.e. the main diagonal) oriented along the vertical crystallographic *c*-axis direction (α = 45° and 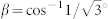). Note that there is no velocity modulation for a clockwise rotation about the *c*-axis, thus indicating that the system is purely transversely isotropic. (e) Fe-*hcp* unit cell and its [0 0 1] fast velocity axis direction. (f) V_p_ of Fe-*hcp* with the fast velocity direction along the vertical Earth's spinning axis (α = β = 0°) showing the 90°-periodicity produced by a clockwise *z*-axis rotation (i.e. α = 0°…180°).

The first selected model phase is the bare Fe-*bcc*, whose unit cell has its fast velocity axis at 54.74° from the Earth's spin axis (see Fig. 3a). Using the polycrystalline aggregate concept and applying the mathematical relations given in the Methods section, we obtain the phase velocity behavior shown in Fig. 3b. On the one hand the starting cubic tensor keeps the cubic symmetry for each clockwise rotation about the *c*-axis, defined by an angle α that varies as α = 90°·*n*, for *n* = 0…4. On the other hand, the stiffness matrix becomes hexagonal every α = (90°·*n* + 45°) degrees of rotation. In our model we assume that the bare Fe-*bcc* phase composes the shallow part of the EIC and that it is always oriented in such a way that the resulting polycrystalline aggregate keeps the cubic symmetry. This particular crystal orientation will approximate the situation where the cooling direction in the Earth's core is predominantly radial (i.e., parallel to the gravity field).

When the reference crystallographic axes of Fe-*bcc* are rotated clockwise by the angle α = 45° around the *c*-axis and the angle β = cos^−1^ (3^−1/2^)° around the *b*-axis, we obtain a positioning such that the main unit cell diagonal is parallel to the Earth's spin axis (Fig. 3c). The elastic stiffness tensor is now that of a perfectly transverse isotropic material over all possible ξ-angle probing directions (Fig. 3d), which means that there exists an infinite-fold axis of rotation. The elastic constants of such a cylindrically averaged *bcc* aggregate (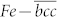) are shown in Table 1. This type of crystal alignment would be stabilized at the shallow part of the EIC if the effects of rotation and convection in the outer core are taken into account^34^, leading to a cylindrical radial heat flux direction.

We further consider the Fe-*hcp* model consisting of a polycrystalline iron aggregate with all [001] crystallographic axes aligned along the Earth's rotational axis (Fig. 3e). The elastic tensor of such an aggregate is always identical to that of the *hcp* single-crystal. However, the V_p_ modeling shown in Fig. 3f clearly indicates that every 90° of clockwise rotation around the *c*-axis, a cylindrical symmetry (i.e., a transverse isotropic velocity model) is achieved, the c_11_ elastic constant being responsible for such a periodicity. It is worth reminding that a hexagonal system is actually one of many symmetry classes of the transversely isotropic system, in which a condition holds that 
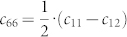
. It is precisely this Fe-*hcp* model phase, specifically oriented by an angle α = 90°·*n*, for *n* = 1…4, that has been chosen as a possible iron component for the EIC. The presence of vertically aligned polycrystalline Fe-*hcp* aggregate at the EIC also boosts the idea that the inner core's heat dissipates along the cylindrical radial direction^34^. We have not considered the Fe-*hcp* with its fast velocity axis in the equatorial plane^7^ because a very few seismological data points would be explained by such a model. Interestingly, the maximum seismic data points matching (minimum misfit) always occurs when the iron crystals have their fast velocity axis oriented as shown in the left panels of Fig. 3. All the others intermediate orientations have very poor seismic data coverage, and therefore were not considered here.

### The *Candy Wrapper* velocity model to explain the EIC hemispherical dichotomy

**Figure 4:**
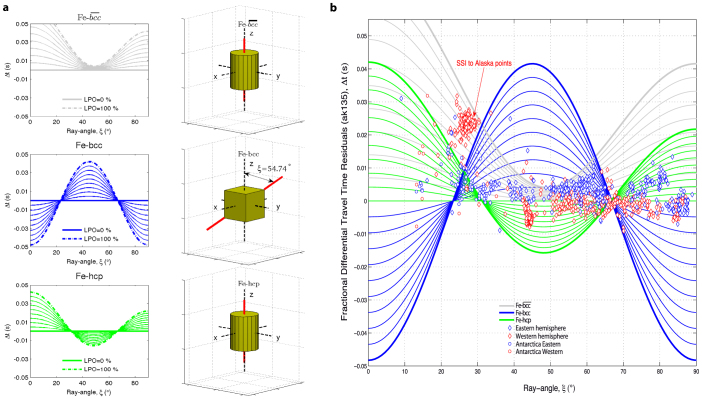
Velocity models versus degree of lattice preferred orientation. Panel (a) shows the three model velocities used in this study as a function of the degree of LPO. Both 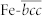 and Fe-*hcp* hold the hexagonal symmetry for every kind of rotations about the *z*-axis, whereas the bare Fe-*bcc *changes from hexagonal to cubic symmetry when the probing angle direction is set along the fast main diagonal. Note that the bare cubic phase (Fe-*bcc*) is the only model that provides exactly the same kind of negative residuals (low velocities) at both polar and equatorial directions. The red vector shows the direction of the fast velocity axis. (b) *Candy Wrapper* shaped velocity model for PKPbc-df data points for a depth probing range of 125.8-345.1 km. Also note that when omitting the data from SSI region, the strength of elastic anisotropy changes drastically from 3% to 0.7%.

**Figure 5:**
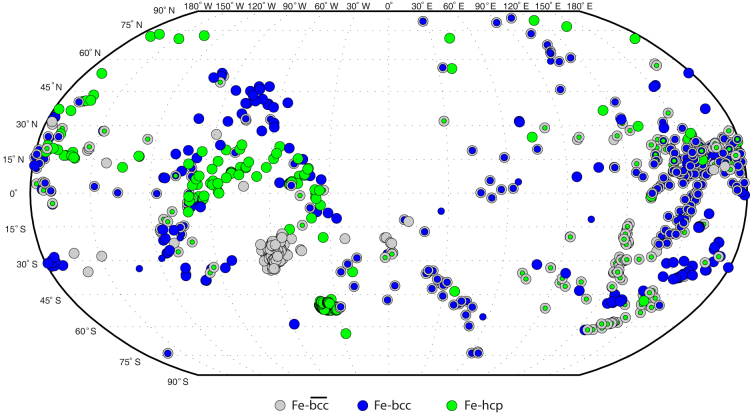
Earth's inner core surface map showing the local distribution of different iron model phases. Each point location corresponds to latitude and longitude of the incoming PKPdf-wave at the inner core surface.

According to Eq. (4) of the Methods section, seismological travel-time data can be directly compared to the proposed iron velocity models to obtain information about EIC anisotropy and composition. In general, the magnitudes of single-crystal elastic constants that are entering into the velocity model are reduced in proportion to the amount of randomly oriented material mixed with the aligned Fe crystals. This effect is taken into account in our *Candy Wrapper* shaped model (Fig. 4) by introducing an LPO variable that considers such an acoustic velocity variation. By selecting the seismological data belonging to the different curves of this complex velocity model, it becomes possible to provide the iron phase map distribution in the uppermost part of EIC (Fig. 5).

A significantly different image emerges for the two hemispheres, with the quasi-western side of the EIC made of well-separated patches of different kinds of iron phases. On the contrary, the quasi-eastern hemisphere appears less discriminating with a spotty-like distribution of points. In this side we cannot uniquely attribute the seismological data points to either one of the three iron phases since it often occurs that two or even all three iron phases can be used to describe the same observed travel-time. As a general tendency, however, the cubic iron is still the dominating phase for the whole EIC, though the hexagonal phase has a remarkable weight in its quasi-western side. To explain this behavior, we make reference to the thermal convection scenario proposed recently by Monnereau *et al.*^21^ and Alboussiére *et al.*^20^, where solidification takes place in the western and melting in the eastern part, giving an overall eastern drift of material. On the basis of annealing experiments, it has been suggested^35^ that the strong solidification texture of newly crystallized iron would be gradually lost during material translation toward the melting side of the EIC. Thus, the crystallizing side (the western hemisphere) would be strongly textured, whereas the melting side (the eastern hemisphere) would be essentially elastically isotropic. This picture is in line with our finding, which shows the quasi-western hemisphere dominated by pure and spatially localized iron phases. Although the process of solidification texturing is more likely to occur if the EIC crystallizes dendritically^34^,^35^, experiments involving non-dendritic solidifications have also shown important solidification texturing^36^. Dendrites usually grow along preferred crystallographic directions and tend to align with the local heat flow^37^. It is exactly this dendritic solidification process, which results from a morphological instability of the solidification front, that provides the needed discriminating force for stabilizing separated domains of Fe-*hcp* and -*bcc* in the western EIC material. Dendritic crystallization conditions are also supported by the existence of constitutional super cooling regions at the ICB^38^,^39^.

The western hemisphere has a slow solidification rate that indeed favors the formation of strongly textured material made of rather small crystals (i.e. more phase boundaries would be present). However, the grain growth process during migration to the melting side can lead to a loss of texturing and to the formation of larger grains. This yields to a more heterogeneous quasi-eastern hemisphere with larger compressional wave velocities. Accordingly, larger (small) grain size and therefore less (more) liquid phase boundaries^40^ are likely to be present in the eastern (western) part providing faster (slower) compressional velocities. Such grain-size and texturing hemispherical variations agree very well with the seismologically observed asymmetry shown in Fig. 2.

A large scatter of differential travel-times observed for the polar PKP paths (relative to the differential travel times corresponding to the equatorial PKP paths) might tentatively be explained by the existence of two distinct polar channels. Recent PKPbc-PKPdf observations from the northern hemisphere to the Antarctic stations^13^ can be assigned to either 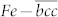, Fe-*bcc* or Fe-*hcp*, thus revealing a kind of an undiscriminating behavior of the iron phases in the quasi-eastern hemisphere. On the contrary, the group of southern Pacific earthquakes observed in the northern hemisphere can only be accounted for by the existence of highly aligned and cylindrically ordered 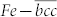 crystals, confined solely in the quasi-western hemisphere. However, more polar paths are needed before it will be possible to draw more sound conclusions.

### Thermodynamic stability scenario of the cubic iron phase

**Figure 6:**
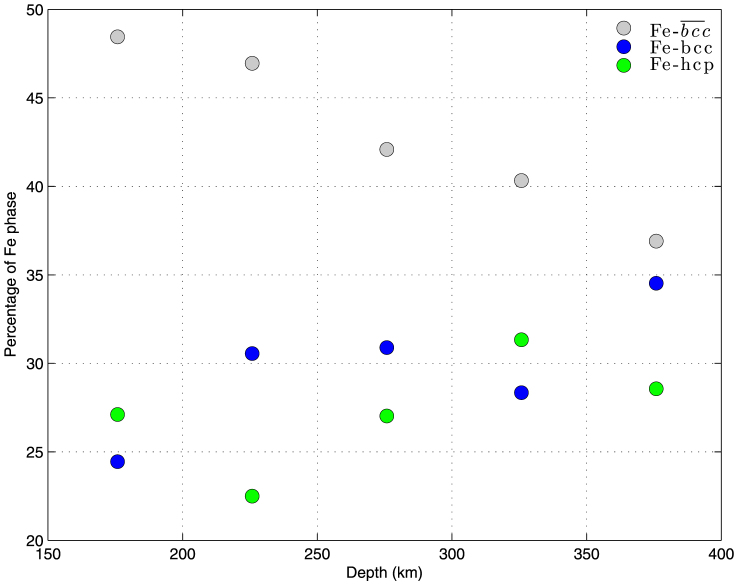
Iron crystal shape stability as a function of P-wave probing depth. Seismic data were binned every 50 km of P-wave penetration depth. Note the almost linear decreasing behavior (positive slope) shown by the 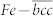 phase, which dominates at the shallower part of the Earth's inner core.

Figure 6 shows the result of binning seismological data every 50 km of P-wave penetration depth. The 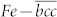 phase is characterized by an almost linearly decreasing percentage, whereas the bare cubic and the hexagonal isomorphs tend to increase their stability with depth. This finding is in line with the fact that the cubic iron phase with a cylindrical symmetry is more stable in the shallow part of the Earth's inner core, especially where hotspots are presents. Strong support for such stability behavior is also provided by a recent work of Geballe *et al*.^41^. Perhaps most surprisingly, we observe that the cubic shaped iron crystal (either as Fe-*bcc* or 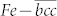) remains the dominant phase for the whole depth-range considered in this study.

The thermodynamic stability scenario of 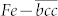 crystals can be revealed by an accurate analysis of the geotherm's evolution inside the solid inner core. The ICB is special for geophysics because at this boundary the liquid and solid iron are in equilibrium (for simplicity we here consider the core to be pure iron). Above this borderline the liquid iron is more stable than solid because the temperature in the core (i.e. the geotherm) is higher than the iron-melting curve. Below the ICB, solid iron is stable because the geotherm is below the iron-melting curve. The temperature gradient in the outer core is slightly less than 0.7 K/km^42^, while the gradient in the solid inner core is likely larger than that, yet the geotherm does not cross the iron melting curve. This means that the temperature in the EIC is extremely close to the melting curve slowly deviating from it since both melting curve and geotherm are continuous curves gradually increasing with pressure. The *bcc* iron phase is likely to have a very narrow temperature field of stability below the melting curve, similar to the case of Xe^9^. Therefore, the geotherm at some pressure above the ICB's pressure might cross the *bcc* stability field and the EIC can then contain the *bcc* phase close to the ICB and an *hcp* deeper down. Considering that the EIC is likely not a pure iron, such a transition will also be gradual due to the changing amount of light elements^43^,^44^. The melting temperatures of *hcp* and *bcc* phases are very close to each other and differ by less than 100 K^29^,^45^. Recent *ab initio* calculations have demonstrated that the stability relation of *hcp* and *bcc* can be further complicated by magnetic effects^45^. As such, small Fe-*bcc* crystals nucleated at the ICB region would align according to the ambient magnetic field during sedimentation if grain rotation is faster than sedimentation's velocity. The magnitude of these magnetic effects, together with crystal size, flow conditions, turbulence and local spatial orientation of the ambient magnetic field could lead to different unit cell orientations (i.e. Fe-*bcc* and 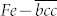)^46^. However, another probable explanation to various lattice orientations can be found in the outward heat flow from the solid inner core to the liquid outer part. Since the heat transfer to the outer core is expected to be more efficient in the cylindrically radial direction, due to the effects of rotation and convection^34^, the *bcc* iron crystals in the shallow EIC would then likely grow by orienting their main diagonals parallel to the Earth's rotational axis. Thus, the transversely isotropic model can be used for modeling the outermost part of EIC, as already demonstrated previously^10^, though it does not hold for larger depths. This is confirmed in our present study. Nonetheless, it is very likely that the observed complex inner core structure arises from distinct local admixture of the two aforementioned mechanisms, which describe the iron crystal alignment as due to either the ambient magnetic field or the directional cooling efficiency.

## Discussion

The achieved results point to a multi-phase EIC iron system, where the cylindrically averaged 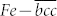 is clearly the dominating phase at the shallow part of the inner core. This supports the idea that heat transport toward the liquid core is more likely to occur in the cylindrically radial direction than with a significant component parallel to the gravity field. Longitudinal hemispherical variations are attributed to different degrees of texturing and grain size as a result of thermal heterogeneities. The western hemisphere can be seen as a conglomerate of dendritically crystallized iron *bcc* and *hcp* domains with different net orientation of fast crystallographic axes. The eastern side instead shows a spotty-like distribution of small iron domains and a much lower degree of crystal shape differentiation due to loss of texturing during the eastern drift of core material. Therefore, the solidifying western hemisphere is made of well-defined and separated anisotropic domains, while the melting eastern side exhibits somewhat random-like distribution of anisotropic materials. This is in excellent agreement with seismological data^14^. A mineral physics explanation to this scenario can be found in a very small difference between the *hcp* and *bcc* melting temperatures, which tends to inhibit the iron shape segregation during the solidification process at the shallow part of the EIC. This is in further agreement with recently proposed geodynamical models^20^,^21^. The crystallization mechanism in the quasi-western hemisphere of the EIC is such that accentuated crystal phase selectivity is achieved with respect to the quasi-eastern hemisphere. This might be attributed to an established dendritic growth mechanism that enhances morphological discrimination between isomorphic iron phases. We here recall that morphology of dendrites strongly depends on the symmetry of the building crystal-unit, and has a special importance for axisymmetric systems such as the hexagonal iron. Therefore, the texture of newly formed crystals in the western hemisphere will be a result of a combination of the local thermal environment plus the texture of the pre-existing solid. Accordingly, large-scale anisotropic regions will then manifest themselves in the solidified western hemisphere material. Conversely, the tightened *hcp*-*bcc* melting temperatures in the eastern hemisphere will result in a similar iron phase stability range, precluding the separation of different phases of iron.

Hence, the introduced *Candy Wrapper* shaped velocity model allows to address the seismologically observed hemispherical dichotomy from a pure mineral physics point of view. The obtained core picture agrees rather well with the recently proposed convective translational model^20^,^21^, thus constituting the basis for a consistent and interdisciplinary EIC scenario. Although we acknowledge the existence of alternative melting/freezing mechanisms^47^,^48^ for the EIC, we were not able neither to endorse nor discard them in this study due to the limited spatial coverage and the type of seismic data available. Nevertheless, further to recent efforts to reconcile dynamical models with seismological observations, a bridge between these two disciplines and mineral physics has now been established.

## Methods

To study the phase composition of the outermost part of the inner-core, we employed both the Transversely Isotropic Model (TIM)^12^,^27^ to different polycrystalline hexagonal aggregates of iron, and the phase velocity model for cubic crystals^49^. Particularly, we consider all the likely stable Fe phases at the EIC conditions (Fe-*hcp*^30^ and Fe-*bcc*^31^) and compare the theoretical and the measured travel-time residuals. The specific orientation of each iron model phase has been selected assuming the existence of two possible kinds of heat flux toward the outer core, that is, one parallel to the gravity field and the other along the cylindrical radial direction.

The key mathematical relation between the observed fractional differential travel-time residuals [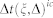] and the theoretical P-wave velocity [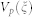] can be achieved through the following equations: 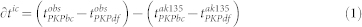

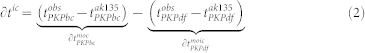

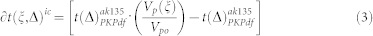

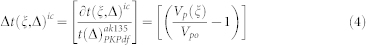
where 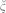 is the ray-angle, 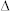 the angular distance from source to receiver, 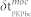 the mantle plus outer-core travel-time anomalies and 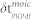 the mantle plus outer and inner-core deviations. The above model assumes that the observed PKPbc and PKPdf differential travel times are minimally effected by Earth structure outside of the EIC due to close proximity of PKPbc and PKPdf ray paths. The longitudinal acoustic velocity of hexagonal and cubic polycrystalline aggregates that enter Eq. (4) can be evaluated employing the set of *ab initio* single-crystal elastic constants (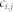) reported in Table 1, and are defined as follows: 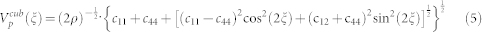

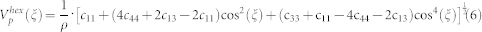
The Hill's averaging method^50^ for both bulk (B_h_) and shear (G_h_) moduli has been applied to obtain the reference P-wave velocity propagation in a polycrystalline media [V_po_ of Eq. (7)], while the spherically symmetric Earth's model *ak135*^25^ has been used to compute theoretical travel-times of PKPdf waves inside the inner-core, 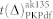. 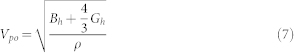


## Author Contributions

M.M., A.B.B. and H.T. designed research and wrote the manuscript. M.M. and A.B.B. developed the mineral physics part and H.T. provided and analyzed the seismological data. All authors (M.M., A.B.B., H.T., E.B., A.U. and R.A.) discussed the results and contributed to revisions.

## Figures and Tables

**Figure 1:**
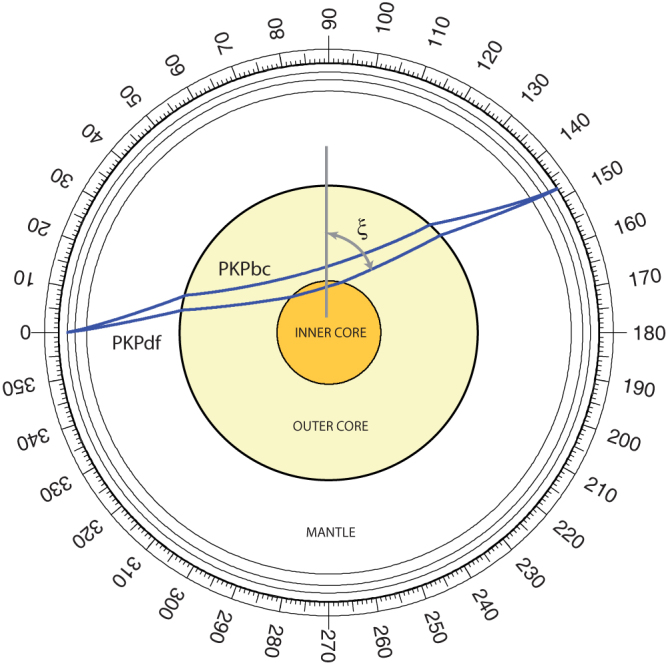
Schematic view of PKP(bc,df) seismic rays for an event at 200 km depth. Geometry of PKPbc and PKPdf ray paths (blue curves) at an epicentral distance of 148°. The ray-angle ξ represents the angle of the incoming PKPdf rays with respect to the vertical Earth's symmetry axis.

**Table 1:** Calculated elastic properties of single-crystal *hcp* and *bcc* iron, and for a cylindrically averaged *bcc* aggregate (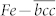). Details about our molecular dynamics calculations can be found in Ref. 10 and references therein. Slightly different *ab initio* elastic constants for Fe phases, both as pure and with light elements, were also obtained by Vočadlo *et al.*^32^,^33^

Parameter	*hcp*	*bcc*	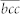
c_11_ (GPa)	1700.4	1561.5	1870.3
c_12_	1251.5	1448.1	1345.2
c_44_	200.1	365.5	159.7
c_13_	1025.0		1242.3
c_33_	1768.7		1973.3
c_66_	224.5		262.6
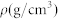	13.543	13.559	
V_po_ (km/s)	10.9667	11.2760	
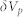 *	0.2955	−0.0138	

*Difference between the *ak135* compressional velocity in the Earth's centre (11.2409 km/s) and the calculated V_po_ value.
